# Healing of an Extensive Periradicular Lesion Subsequent to a Proper Endodontic Treatment of a Mandibular First Premolar with Complex Anatomy

**DOI:** 10.1155/2013/972093

**Published:** 2013-12-08

**Authors:** Hengameh Ashraf, Payam Paymanpour, Maryam Mojtahed Bidabadi, Reihaneh Hajrezai

**Affiliations:** ^1^Department of Endodontics, Shahid Beheshti University of Medical Science, Tehran, Iran; ^2^Department of Orthodontics, Dental Branch, Islamic Azad University, Tehran, Iran

## Abstract

Long-term success of endodontic treatment is strictly dependent on proper shaping and cleaning of the root canal system followed by obturation of entire prepared space. Anatomical variations should be considered during radiographic and clinical evaluation as parts of endodontic treatment. A mandibular premolar with three canals is quite rare and such a tooth requires special canal preparation and obturation techniques. An astute clinician should identify different canal configurations and treat them endodontically well, because presence of an untreated canal could be a reason for failure of endodontic treatment. This paper describes the conventional orthograde endodontic therapy on an unusual mandibular first premolar with three root canals.

## 1. Introduction

A thorough knowledge of anatomy and morphology of root canal system is essential for a successful root canal treatment. Bear in mind that parallel buccolingual radiographs may not always determine correct morphology of tooth. A two-dimensional radiographic view of a three-dimensional tooth, poses serious problems for anatomical assessment of a root canal system that need to be treated. Different horizontal angulations of X-ray cone are considered worthwhile adjuncts to clarify hidden aspects of root canal system. Because of anatomical variations, mandibular premolars are difficult for endodontic treatment and a higher failure rate is expected. As we know, main purpose of endodontic therapy is to prevent or resolve periradicular periodontitis. Periradicular radiolucencies develop depending on the balance between microbial virulence factors and host defenses [1]. A primary aim of all endodontic procedures, specially cleaning and shaping, is to remove infective microorganisms and necrotic tissues [2]. Considering that canals are often underprepared in apical one-third [3] and root canal systems cannot be completely disinfected [4–7], obturation of the prepared root canal space is necessary. Obturation reduces coronal leakage and bacterial recontamination, seals the apex from periapical tissue fluids, and entombs remaining irritants within canal [8]. Division of canals into two or more is a challenging situation for both cleaning shaping and obturation of root canal system that could result in periradicular periodontitis. Clinical exploration is essential to detect division of canal. During formation of the root, the apical foramen of each root has a wide opening limited by the epithelial diaphragm and the shape of the pulp canal is like a wide-open tube [9]. Differentiation of a root into separate canals occurs by continued deposition of dentin [10]. A higher number of extra canals in mandibular premolars of African descent have been reported compared with Caucasian patients [11]. International studies have shown the existence of second or third canal was noted in only 23.1 per cent of first mandibular premolars and in only 12.1 per cent of second mandibular premolars [12].  Table 1 describes some studies and case reports which reported mandibular first premolar with three canals.

## 2. Case Report

**Figure 1:**
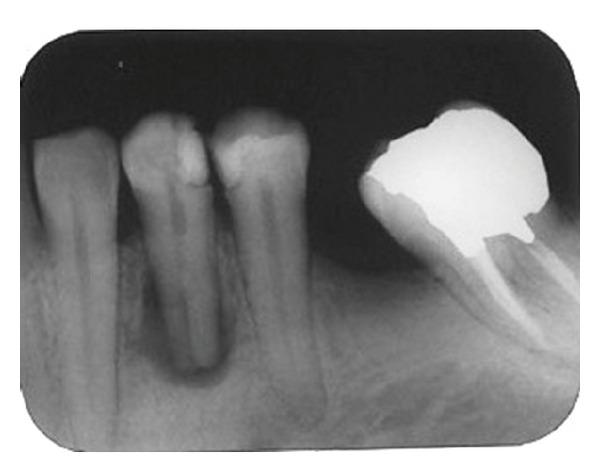
Periapical radiograph of mandibular left first premolar revealing complex root canal system.

A 42-year-old female with no remarkable medical history was referred to the Endodontics Department of Shahid Beheshti Dental School for root canal treatment of her left first mandibular premolar. The patient had not any complaint about the mentioned tooth and the tooth was asymptomatic. However, an extensive radiolucency associated with periradicular lesion of tooth was detected through routine radiographs (Figure 1). There was no sensitivity to percussion. Vitality testing was negative for the tooth suggesting pulp necrosis. A large defective composite resin coronal restoration was present. Periodontal probing was within the normal limits. 

**Figure 2:**
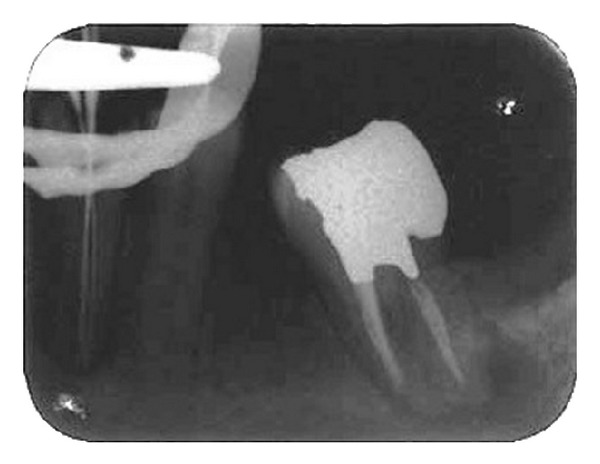
Working length radiograph confirming three root canal orifices.

**Figure 3:**
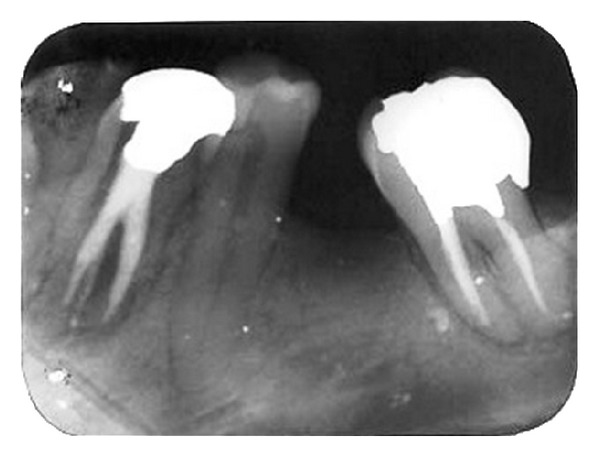
Radiograph revealing complete obturation.

**Figure 4:**
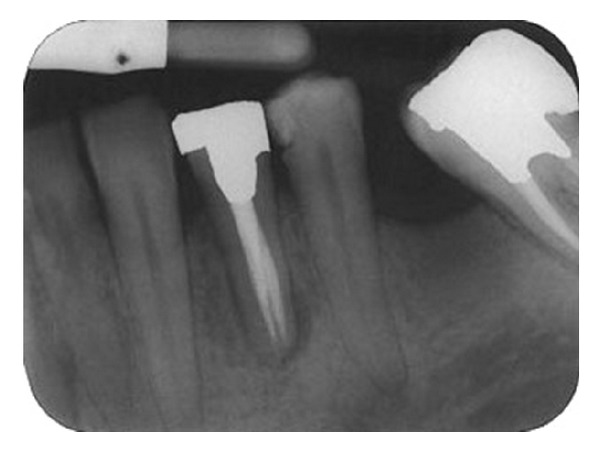
A 3-month recall shows dramatic healing.

**Figure 5:**
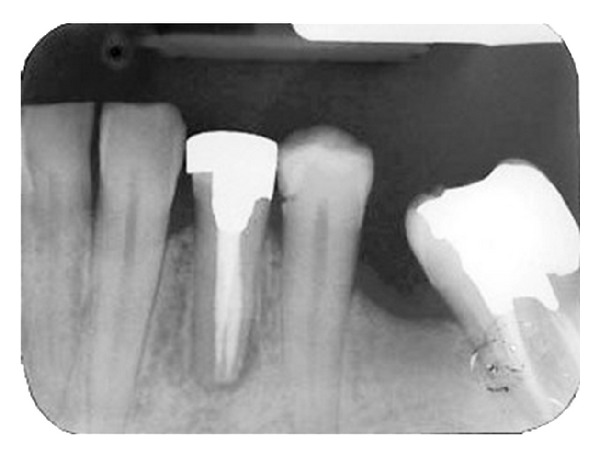
A 1-year recall shows complete healing.

Parallel diagnostic radiograph showed a substantial periradicular lesion, calcification, and division of root canal system at midroot. In this radiograph, an extra canal was obviously diverged distally from the main canal with a sharp angle. 2% Lidocaine plus 1 : 80,000 epinephrine (Darou Pakhsh Co., Tehran, Iran) was used for inferior alveolar nerve block. Rubber dam was applied and access cavity preparation was accomplished. After removing coronal pulpal tissue, only one orifice was observed. Size #2, #3, and #4 Gates Glidden burs (MANI Inc., Japan) were used for initial opening of canal orifice. Through further exploration with a bended #10 and #15 K-files (Maillefer, Switzerland) entrance of divided canals was located. Root canal lengths were estimated with an electronic apex locator (Smarpex, Meta, Korea) and these lengths were confirmed by further radiographs. In this radiograph, trunk of canal seemed to trifurcate at midroot level giving rise to three separate canals and apical foramina (Figure 2). Sodium hypochlorite (5.25%) was used as endodontic irrigant. Apical preparation was performed by using both hand K-files and ProTaper rotary files (DENTSPLY, Switzerland). During preparation, size #15 K-file was used to establish and maintain apical patency. Master apical file was of size #30 for all the root canals. Calcium hydroxide was used as intracanal medicament and access cavity was sealed with temporary filling (Coltosol, Ariadent, Iran). Two weeks later, patient returned for second visit and the tooth was asymptomatic. After application of dental rubber dam, coronal temporary filling was removed. 5.25% sodium hypochlorite was used for removing intracanal calcium hydroxide and root canal irrigation. After drying with paper points, canals were obturated with gutta-percha and AH26 Sealer (DENTSPLY Caulk) via cold lateral compaction technique and access cavity was sealed with temporary filling (Cavit 3M ESPE, St. Paul, MN, USA) (Figure 3). Final radiographic examination showed distal canal diverged from main canal more coronally than mesial canal which divided into two distinct canals at apical one third. At 3-month recall, patient was asymptomatic (Figure 4) and 6-month recall radiograph showed significant healing (Figure 5).

## 3. Discussion

Knowledge of common root canal morphology and its frequent variations is a prerequisite for successful endodontic treatment. These variations in pulpal anatomy must be considered at the beginning of endodontic treatment [17]. The possibility that there is more than one root canal in lower premolar teeth should always be expected. Accurate straight and angled preoperative radiographs and clinical clues are essential in exploring the number of roots canals that exist in a tooth. In this case to view individual canals we used 35-degree distal angled radiograph which could separate canals. Presence of untreated canal may be a reason for failure of endodontic therapy [18]. Electronic apex locators are useful adjuncts for determining working length in all cases, particularly complex anatomical variations. Use of both of electronic devices and angled radiographs provides greater accuracy [19]. Missing of anatomic information may be resulted if electronic apex locators are used exclusively. Additional anatomic information about root canals can be obtained by careful observation of positioning and deformation of instruments [20]. It is most important to be on the lookout for additional canals. In this case, a single broad root canal trifurcated into three separate root canals. Enhanced lighting and visibility are important aids for locating root canals. Dental operating microscope provides magnification and illumination which improves the ability to locate and negotiate canals [21]. When working with operating microscope can many times see hypochlorite bubbling in extra canal, marking its presence. On occasion, dyes or transillumination may be helpful in locating additional canal [22].

Although no significant difference was found in periapical healing between one- or two-visit endodontic treatment of necrotic teeth [23], some factors such as the patient's health, anxiety, fatigue, and symptoms, in addition to complexity of treatment, should be considered. Su et al. described that the healing rate of single- and multiple-visit root canal treatment is similar for infected teeth [24]. Multiple-visit and single-visit root canal treatment demonstrated almost equal success but most important aspect for success in treatment is indication of each case and then its subsequent treatment, be it multiple or single visit root canal treatment [25].

Nonsurgical endodontic therapy proved successful in promoting the healing of periapical lesions; irrespective of the size of the lesion every attempt should be made to treat the periapical lesions with non surgical endodontic therapy [26]. Intracanal placement of calcium hydroxide has been proposed because of its antimicrobial properties in necrotic cases that cannot be treated in one visit [27]. It has been reported that treatment with calcium hydroxide resulted in high frequency of periapical healing and some lesions, especially in young patients, were reduced or had completely disappeared only 1 or 3 months after treatment [28]. Ca(OH)_2_ is proven to assist periapical healing by virtue of its antibacterial properties and also by its biological action facilitating osseous repair [29].

Finally, there is a need for further studies to determine the frequency of anatomic variations in root canal system of Iranian population and referral to an endodontist seems to be mutually beneficial in complex anatomic variations of root canal system.

## 4. Conclusion

Although frequency of first mandibular premolars with an unusual anatomical configuration is not common, each tooth should be investigated carefully, clinically, and radiographically to detect possible anatomical variations before commencement of endodontic treatment.

## Figures and Tables

**Table 1:** Trifurcated canals for the mandibular first premolar (%).

Zillich and Dowson [12]	4.3
Yoshioka et al. [13]	0
Sert and Byirli [14]	2.2
Al-Qudah and Awawdeh [15]	Case report
Nallapti [16]	Case report
